# Utility of [^18^F] Fluoro-Deoxyglucose Positron Emission Tomography/Computed Tomography for Staging and Therapy Response Evaluation in Pediatric Rhabdomyosarcoma: A Case Series and Literature Review

**DOI:** 10.3389/fmed.2020.00281

**Published:** 2020-07-14

**Authors:** Ri Sa, Danyan Liu, Hongguang Zhao, Sen Hou, Qiuyu Lin, Feng Guan

**Affiliations:** ^1^Department of Nuclear Medicine, The First Hospital of Jilin University, Changchun, China; ^2^Department of Radiology, The First Hospital of Jilin University, Changchun, China

**Keywords:** PET/CT, rhabdomyosarcoma, standardized uptake value (SUV), metabolic tumor volume (MTV), total lesion glycolysis (TLG)

## Abstract

**Background:** The role of [^18^F] fluoro-deoxyglucose [[^18^F] FDG] positron emission tomography (PET)/computed tomography (CT) in pediatric rhabdomyosarcoma (RMS) is not well-established. This manuscript explores the role of staging and therapy response evaluation of PET/CT in a series of patients with RMS.

**Methods:** Thirteen consecutive patients with pathologically proven RMS underwent baseline PET/CT scan and a second PET/CT for evaluation of therapy response. Maximum standardized uptake value (SUV_max_), mean standardized uptake value (SUV_mean_), highest standardized uptake peak value (SUV_peak_), metabolic tumor volume (MTV), and total lesion glycolysis (TLG) were obtained from baseline PET/CT and were used as potential predictors for evaluation of metabolic treatment response.

**Results:** On baseline PET/CT, most RMSs are located in the pelvic cavity, and upper arms ranked second. The primary lesions were large and showed invasion to the surrounding tissues. Lymph node metastases were seen in eight patients, and eight patients showed distant metastasis to the lung, liver, and bone. The median SUV_max_, SUV_mean_, and SUV_peak_ of primary sites were 7.1, 4.0, and 5.9, respectively. The median MTV and TLG were 196.6 cm^3^ and 780.2, respectively. After therapy, six patients received complete metabolic response (CMR) and non-CMR occurred in seven patients on the second PET/CT. SUV_max_, SUV_peak_, MTV, and TLG in patients with CMR were significantly lower than those in patients with non-CMR.

**Conclusions:** Primary sites and metastatic lesions of RMS demonstrate increased glycolytic activity, which may allow them to be imaged using [^18^F] FDG PET/CT. Metabolic parameters derived from the baseline PET/CT have potential value for predicting CMR to therapy in pediatric RMS.

## Introduction

Rhabdomyosarcoma (RMS) is the most common soft tissue sarcoma in children and adolescents, but the disease is still rare, with about 400 newly diagnosed cases each year in Europe and a similar incidence in the USA (1). RMS is a high-grade, malignant neoplasm in which the current therapy strategies for RMS are multimodal approach, comprising surgical resection, chemotherapy, and/or radiation therapy in order to control the primary tumor to the utmost (2). The chance of cure with widely metastatic and recurrent disease of RMS is very low, and patients experience months of intensive, multifaceted therapies that can bring life-threatening acute toxicities and, in some cases, life-changing late effects (3).

The potential factors which are associated with ultimate outcome of RMS are controversial (4, 5). Nevertheless, it is well-accepted that the favorable status at the end of therapy is crucial to promising outcome of RMS. By contrast, residual mass in patients with RMS at the end of therapy had a significant impact on failure-free survival (6). Thus, identifying predictors that can select therapy-sensitive tumors is considered as an important step to manage treatment strategies to avoid poor outcome. [^18^F] fluoro-deoxyglucose [[^18^F] FDG] positron emission tomography (PET)/computed tomography (CT) is one of the most advanced multimodal techniques available. [^18^F] FDG PET/CT can accurately detect the extent and metabolic activity of tumor lesions in patients with malignant tumors, aiding staging and evaluation of therapy response in many malignant tumors. However, to date, only several retrospective studies qualitatively interpreted the images to demonstrate the potential role of [^18^F] FDG PET/CT in RMS but failed to include volumetric [^18^F] FDG uptake measurements such as metabolic volume and lesion glycolysis. Therefore, the aim of this study was to demonstrate the utility of metabolic parameters derived from baseline PET/CT in staging and evaluation of therapy response of RMS.

## Patients and Methods

### Patients

We enrolled the patients with the following inclusion criteria: histopathologically confirmed RMS from January 2008 to November 2018; under age of 18 years old. Those with other malignant tumors beyond RMS were excluded.

### [^18^F] Fluoro-deoxyglucose PET/CT

Baseline PET/CT was performed before therapies, and the second PET/CT for evaluating therapy response was performed within 4 weeks after antitumor therapy. PET/CT scans including semiquantitative and metabolic volumes measurement were reviewed by two nuclear physicians who were unaware of the results of any other imaging tests and the clinical data by consensus.

Patients were asked to fast for 6 h. [^18^F] FDG (4.07 MBq/kg) with a radiochemical purity of >95% (Sumitomo Corporation, Japan) was given through intravenous injection, and the patients were asked to rest for approximately 60 min after the injection. Four children (age ≤ 3 years old) who could not give collaboration during the tests were sedated after injection of [^18^F] FDG immediately prior to commencement of imaging. The PET/CT scan was performed using a hybrid PET/CT scanner (Biograph 16HR; Siemens, Germany). Here, all [^18^F] FDG PET/CTs were performed with a standard acquisition and reconstruction protocol. Quality assurance and quality control procedures for the PET system were carried out accurately on a daily basis. The scan range was from the skull to the upper part of mid-thigh or feet. A low-dose CT protocol (100 mAs, 140 kV, tube rotation time of 0.5 per rotation, pitch of 6, slice thickness of 5 mm, and shallow breathing) was first applied and followed by the PET scan (3-min emission scan per table position).

In each examination, the representative lesion was defined as a single lesion with the highest FDG uptake, even though patients had multiple active lesions. A region of interest (ROI) was drawn around the representative lesion with boundaries drawn to include the lesion in transaxial, coronal, and sagittal views. Maximum standardized uptake value (SUV_max_) was calculated from a single voxel exhibiting the SUV_max_ within a representative lesion. Mean standardized uptake value (SUV_mean_) was the mean value of voxels within the ROI. Highest standardized uptake peak value (SUV_peak_) was the SUV_mean_ of a 1-cm^3^ three-dimensional ROI showing the highest value in the representative lesion. They were obtained in the same area as the pretreatment lesion in case of no abnormal uptake after treatment. Metabolic tumor volume (MTV) and total lesion glycolysis (TLG) were calculated as volumetric parameters in the representative lesion as well as in whole-body lesions. MTV was defined as the volume showing abnormal FDG uptake greater than any parts of the liver in this study. TLG was calculated as the product of MTV multiplied by the SUV_mean_. The metabolic parameters were measured on the Siemens MIWP workstation (Syngo MIWP; Siemens Medical Solutions, Erlangen, Germany).

### Therapy Response Assessment

The SUV_max_, SUV_mean_, SUV_peak_, total MTV, and total TLG were determined as metabolic PET parameters for each patient. The second PET/CT scans were used to classify therapy response into four categories according to the European Organization for Research and Treatment of Cancer (EORTC) criteria: complete metabolic response (CMR, no FDG uptake within the tumor volume), partial metabolic response (PMR, SUV_max_ reduction >25% after treatment), stable metabolic disease (SMD, SUV_max_ increase or decrease <25%), progressive metabolic disease (PMD, SUV_max_ increase >25% or increase in the extension of tumor uptake >20% in the longest dimension or the appearance of new FDG uptake) (7). Patients with PMR, SMD, and PMD were then grouped as non-complete metabolic responders (non-CMR).

### Statistical Analysis

Statistical analysis was performed using SPSS software (version 22; IBM). The continuous variables with non-normal distribution are presented as median (range). The categorical variables are reported as number (percentage). The factors that may have affected CMR in RMS were analyzed. Fisher exact tests and χ^2^ tests were used to test the significance of categorical data such as gender (male or female), tumor size (<5.0 cm or ≥5.0 cm), tumor margin (T1, confined to anatomic site of origin, or T2, extension and/or fixative to surrounding tissue), lymph node metastases (yes or no), distant metastases (yes or no), and therapy (chemotherapy only or surgery/RT+ chemotherapy). The non-parametric Mann–Whitney *U*-test was used to compare quantitative data when it was not normally distributed including age, SUV_max_, SUV_mean_, SUV_peak_, MTV, and TLG. All *p*-values were two-sided, and *p* < 0.05 was considered statistically significant in all analyses.

### Literature Search

We performed a PubMed/Medline search by using MeSH terms focusing on articles on RMS-related and RMS-specific infections and on use of nuclear imaging with [^18^F] FDG PET/CT scans. Basic information was collected, including author, journal, year published, and number of patients. Specific data were collected, including the study purpose, recorded parameters, and main findings of PET or PET/CT.

## Results

Thirteen consecutive patients with newly diagnosed RMS in our department were enrolled. Clinical characteristics, therapy regimen, treatment response, and outcome of all patients with RMS are shown in Table 1. The findings of baseline [^18^F] FDG PET/CT are shown in Table 2.

**Table 1:** Clinical characteristics, treatment response, and outcome in the 13 patients with rhabdomyosarcoma.

**No**.	**Age**	**Gender (F/M)**	**Pathological type**	**Clinical symptom**	**Therapy**	**Therapy response**	**Follow up time**	**Outcome after the second PET/CT**
1	11 y	F	ERMS	Chest pain	RT, chemotherapy	CMR	3.4 y	Observation
2	1 y	M	ERMS	Arm pain	Surgery, chemotherapy	CMR	1.5 y	Observation
3	2 mo	F	ERMS	Facial mass	RT, chemotherapy	SMD	13 mo	Therapy changed, but the patient died
4	7 y	M	ERMS	Abdomen pain	Surgery, chemotherapy	CMR	9 mo	Observation
5	3 y	M	ERMS	Abdomen pain	Chemotherapy	CMR	17mo	Observation
6	3 y	M	ERMS	Mass in hips	Surgery, chemotherapy	CMR	21 mo	Observation
7	12 y	F	ERMS	Mass in toe	RT, chemotherapy	PMR	13 mo	Observation
8	8 y	F	ERMS	Abdomen pain	RT, chemotherapy	PMR	6 mo	Therapy prolonged
9	13 y	M	ARMS	Arm pain	Chemotherapy	PMR	7.2 mo	Observation
10	4 y	M	ERMS	Abdomen pain	RT, chemotherapy	PMD	5.5 mo	Therapy changed
11	4 y	F	ERMS	Abdomen pain	RT, chemotherapy	PMD	6.5 mo	Therapy changed, but the patient died
12	5 y	F	ERMS	Axillary mass	Surgery, chemotherapy	CMR	7.4 mo	Observation
13	11 y	M	ERMS	Mass in hips	Surgery, chemotherapy	SMD	6 mo	Therapy prolonged

*y, year; mo, month; F, female; M, male; ARMS, alveolar rhabdomyosarcoma; ERMS, embryonal rhabdomyosarcoma; RT, radiation therapy; CMR, complete metabolic response; PMD, progressive metabolic disease; SMD, stable metabolic disease; PMR, partial metabolic response*.

**Table 2:** Findings of baseline PET/CT in the 13 patients with rhabdomyosarcoma.

**No**.	**Primary site**	**Size**	**SUV_**max**_**	**SUV_**mean**_**	**SUV_**peak**_**	**MTV (cm^**3**^)**	**TLG**	**T**	**Lymph node metastases**	**Distant metastases**
1	Mediastinum	b	4.9	3.4	4.1	77.2	229.4	T2	Neck, mediastinum, hilum	Liver, sternum
2	Upper arm	b	5.4	3.8	3.4	37.9	112.3	T1	-	-
3	Maxillofacial	b	7.1	4.3	6.2	25.0	114.1	T2	-	Ilium
4	Intraperitoneal	a	7.2	4.6	5.6	196.6	780.2	T1	-	-
5	Pelvic	b	7.1	4.0	5.9	40.8	160.8	T2	-	Lung
6	Hips	a	4.3	2.6	3.7	13.7	42.6	T1	-	-
7	Toes	a	6.4	3.7	5.9	824.8	2969.3	T2	Neck, mediastinum, hilum, abdomen, pelvic	Rib, ilium
8	Pelvic	b	9.0	4.9	6.4	556.4	3115.8	T2	Neck, mediastinum, abdomen, pelvic	Lung
9	Upper arm	b	9.2	4.3	6.9	264.6	1202.5	T2	Neck, mediastinum, axillary, elbow, hilum	Humerus
10	Pelvic	b	15.0	7.3	9.1	496.7	4023.3	T2	Mediastinum, abdomen, pelvic	Rib, spine, ilium
11	Pelvic	b	12.9	6.9	7.6	319.3	2362.8	T2	Abdomen, pelvic	Lung
12	Axillary	b	3.9	2.4	3.4	46.1	121.4	T2	Axillary	-
13	Hips	b	7.0	3.4	5.5	402.9	1814.1	T2	Pelvic, inguen	-

*Size: a, < 5 cm in diameter; b, ≥ 5 cm in diameter; T, tumor; T1, confined to anatomic site of origin; T2, extension and or/fixative to surrounding tissue; -, no metastatic lesions*.

*SUV_max_, maximum standardized uptake value; SUV_mean_, mean standardized uptake value; SUV_peak_, standardized uptake peak value; MTV, metabolic tumor volume; TLG, total lesion glycolysis*.

The age of the 13 patients ranged from 2 months (case 3) to 13 years (case 9), and six of them were female. The diagnosis was established by ultrasonography (US)-guided fine-needle aspiration in all patients before baseline PET/CT. Only one patient (case 9) was histologically confirmed as alveolar RMS (ARMS).

On baseline PET/CT, RMS was most commonly located in the pelvic cavity, while upper arms ranked second. The primary lesions in the majority of patients (except cases 4, 6, and 7) were large, in which the diameter were larger than 5 cm. Primary sites in three patients (cases 2, 4, and 6) were confined to the anatomic site of origin, while primary sites in other patients demonstrated invasion to the surrounding tissues. Lymph node metastases were seen in eight patients (cases 1 and 7–13) while cases 1, 3, 5, and 7–11 showed distant metastasis to the lung, liver, and bone. The median SUV_max_, SUV_mean_, and SUV_peak_ of target lesions were 7.1 (3.9–15.0), 4.0 (2.4–7.3), and 5.9 (3.4–9.1), and the median MTV and TLG were 196.6 (13.7–824.8) cm^3^ and 780.2 (42.6.1–4,023.3), respectively.

**Figure 1:**
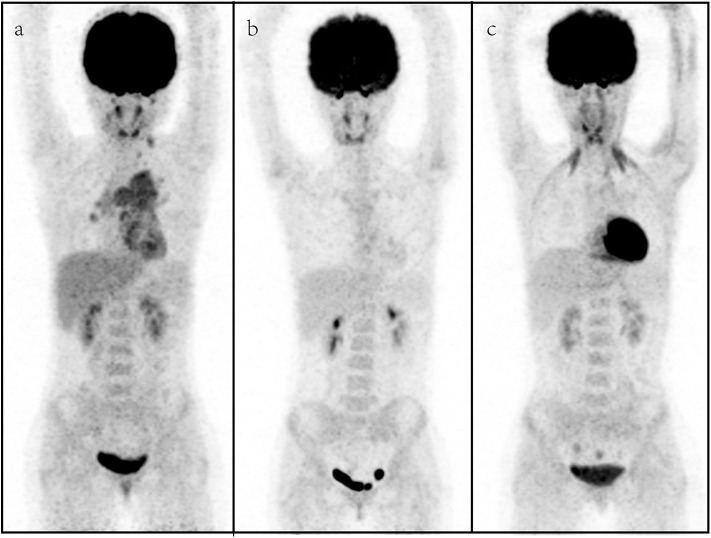
A representative case of an 11-year-old female with rhabdomyosarcoma. Baseline maximum intensity projection (MIP, **a**), showing hypermetabolic disease in laterocervical, mediastinal, and hilar lymph nodes, liver, and sternum [maximum standardized uptake volume (SUV_max_): 4.9, SUV_mean_: 3.4, SUV_peak_: 4.1, metabolic tumor volume (MTV): 77.2 cm^3^, total lesion glycolysis (TLG): 229.4]. PET/CT **(b)** after therapy showing a complete metabolic response with no fluoro-deoxyglucose (FDG) uptake in the previous sites. PET/CT **(c)** for follow-up revealed no FDG uptake lesions.

**Figure 2:**
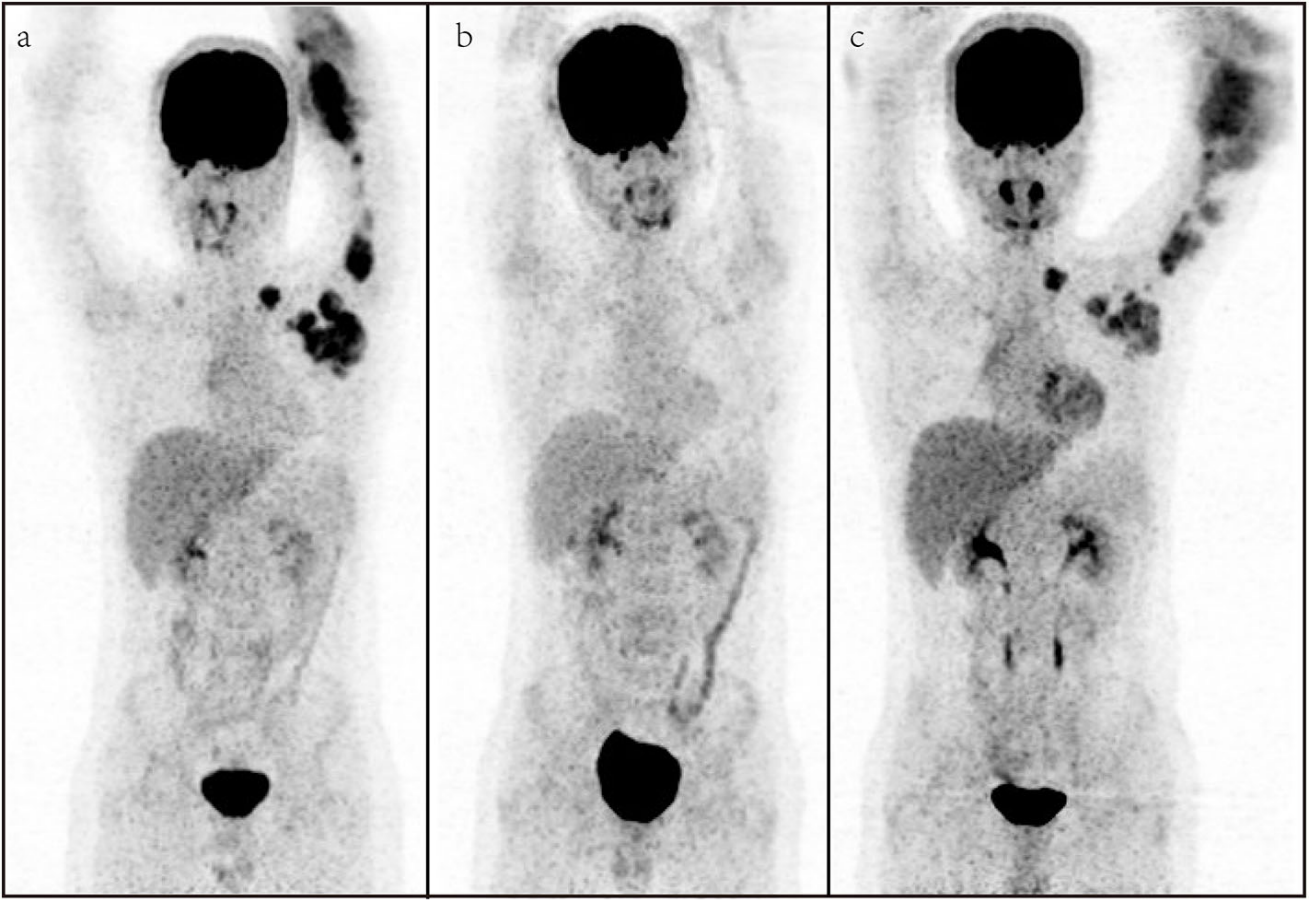
A representative case of a 13-year-old male with rhabdomyosarcoma. Baseline maximum intensity projection (MIP, **a**), showing hypermetabolic disease in laterocervical, mediastinal, axillary, elbow, and hilar lymph nodes and humerus [maximum standardized uptake volume (SUV_max_): 9.2, SUV_mean_: 4.3, SUV_peak_: 6.9, metabolic tumor volume (MTV): 264.6 cm^3^, total lesion glycolysis (TLG): 1,202.5]. PET/CT **(b)** after therapy showing a partial metabolic response with fluoro-deoxyglucose (FDG) uptake lymph nodes in the axillary. PET/CT **(c)** for follow-up showing metabolic progression of disease with appearance of new lesions.

Five patients received surgery before the chemotherapy, while six patients received radiation therapy (RT) before the chemotherapy. After the therapy, all patients received the second PET/CT for evaluation of metabolic treatment response, in which six patients achieved CMR, and non-CMR occurred in seven patients (PMR in three patients, SMD in two patients, and PMD in two patients) on the second PET/CT. All patients with CMR and two patients with PMR on the PET/CT turned to observation, and other patients prolonged or changed the therapy. The median follow-up time was 9.0 months, and two patients (cases 3 and 11) died. The results of [^18^F] FDG PET/CT of cases 1 and 9 are shown in Figures 1, 2, which were confirmed as CMR and PMR at the second PET/CT, which were performed for evaluation of therapy response, while the two patients received the third PET/CT during the follow-up while case 1 obtained CMR and case 9 suffered from PMD.

Comparisons of the clinical characteristics and metabolic parameters derived from baseline PET in RMS patients regarding therapy response (CMR and non-CMR) are shown in Table 3. SUV_max_, SUV_peak_, MTV, and TLG in patients with CMR were significantly lower than those in patients with non-CMR.

**Table 3:** Comparison of clinical characteristics and metabolic parameters derived from baseline PET in rhabdomyosarcoma patients regarding to the therapy response (*N* = 13).

**Patients**	**Therapy response**		***Z/x*^**2**^ value**	***p***
	**CMR (*n* = 6)**	**Non-CMR (*n* = 7)**		
Age-median (range)	4 (1–11 y)	8 (2.0 mo−13 y)	−1.076	0.282
Gender-*n* (%)			0.737	0.592
F	2 (33.3%)	4 (57.1%)		
M	4 (66.7%)	3 (42.9%)		
Size-*n* (%)			0.660	0.559
<5.0 cm	2 (33.3%)	1 (14.3%)		
≥5.0 cm	4 (66.7%)	6 (85.7%)		
SUV_max_-median (range)	5.2 (3.9–7.2)	9.0 (6.4–15.0)	−2.217	0.027
SUV_mean_-median (range)	3.6 (2.4–4.6)	4.3 (3.4–7.3)	−1.791	0.073
SUV_peak_-median (range)	3.9 (3.4–5.9)	6.4 (5.5–9.1)	−2.650	0.008
MTV-median (range, cm^3^)	43.5 (13.7–196.6)	402.9 (25.0–824.8)	−2.286	0.022
TLG-median (range)	141.1 (42.6–780.2)	2362.8 (114.1–4023.3)	−2.429	0.015
T –*n* (%)			4.550	0.070
T1	3 (50.0%)	0 (0%)		
T2	3 (50.0%)	7 (100%)		
Lymph node metastases-*n* (%)			3.745	0.103
Yes	2 (33.3%)	6 (85.7%)		
No	4 (66.7%)	1 (14.3%)		
Distant metastases-*n* (%)			3.745	0.103
Yes	2 (33.3%)	6 (85.7%)		
No	4 (66.7%)	1 (14.3%)		
Therapy			0.014	0.906
Chemotherapy only	1 (16.7%)	1 (14.3%)		
Surgery/RT+ chemotherapy	5 (83.3%)	6 (85.7%)		

*T1, confined to anatomic site of origin; T2, extension and or/fixative to surrounding tissue*.

*F, female; M, male; T, tumor; T1, confined to anatomic site of origin; T2, extension and or/fixative to surrounding tissue; SUV_max_, maximum standardized uptake; SUV_mean_, mean standardized uptake value; SUV_peak_, standardized uptake peak value; MTV, metabolic tumor volume; TLG, total lesion glycolysis; RT, radiation; CMR, complete metabolic response*.

## Discussion

To the best of our knowledge, this is the first study to distinguish CMR from non-CMR in therapy of RMS by testing various metabolic parameters derived from the baseline PET, including SUV_max_, SUV_mean_, SUV_peak_, MTV, and TLG, which would assist the management of treatment and avoid poor outcome. In the current literature, we found several studies have only focused on SUV_max_ to reveal the role of [^18^F] FDG PET/CT in the field of diagnosis, initial staging, and predicting prognosis of RMS. The findings of these studies are summarized in Table 4.

**Table 4:** Overview of published studies on pediatric rhabdomyosarcoma and [^18^F] FDG PET/CT.

**Study**	**Journal**	**Year**	**Image**	**Patients number**	**Purpose**	**Method**	**SUV_**max**_ of primary sites**	**Findings**
Ben Arush MW et al. (10); Ricard et al. (11); Dong et al. (19)	J Pediatr Hematol Oncol Clin Nucl Med; Q J Nucl Med Mol Imaging	2006; 2011; 2012	PET or PET/CT	3 cases; 13 cases; 28 cases	Staging and restaging	SUV_max_	-; 3.7 (range,2.0–6.9); 6.0 (range, 1.4–22.6)	PET/CT can be useful in staging and restaging pediatric RMS, especially for assessing lymph nodes and bone involvement, and for detecting unknown primary sites of RMS, with potential therapeutic strategy alteration.
Klem et al. (20)	J Pediatr Hematol Oncol	2007	PET/CT	31 cases	Staging	Comparison with CIMs; SUV_max_	6.4 (range, 2.4–12.7)	CIMs was equivocal with PET for the detection of regional or distant spread. Comparing to the final clinical determination of disease, PET was 77% sensitive and 95% specific.
Federico et al. (9); Eugene et al. (17); Tateishi et al. (12); Völker et al. (21)	Pediatr Blood Cancer; Nucl Med Commun; Ann Nucl Med;J Clin Oncol	2013; 2012; 2009;2007	PET/CT; PET	17 cases; 13 cases; 35 cases; 12 cases	Staging	Comparison with CIMs; SUV_max_	7.2 (range, 2.5–19.2); 6.2 ± 3.8 (range, 2.7–15.4); –; 7.0 ± 3.4	PET/CT performed better than CIM in identifying nodal, bone, bone marrow, and soft tissue disease in children with RMS. CIM remains essential for detection of pulmonary nodules.
Dharmarajan KV et al. (18)	Int J Radiat Oncol Biol Phys	2012	PET	97 cases	Predicting local control	SUV_max_	7.0 (range, 0–31)	Negative postradiation PET predicted improved LRFS. Negative baseline and preradiation PET findings suggested statistically insignificant trends toward improved LRFS.
Casey DL et al. (22)	Int J Radiat Oncol Biol Phys	2014	PET	107 cases	Predicting outcome	SUV_max_	8.1 (range, 0–22)	The baseline SUV_max_ (<9.5 vs. ≥9.5) was predictive of PFS and OS, but not LC. The SUV_max_ after induction chemotherapy (<1.5 vs. ≥1.5) was similarly predictive of PFS and was associated with LC and OS. A positive PET after local therapy was predictive of worse PFS, LC, and OS.
El-Kholy et al. (23); Baum et al. (24)	Nucl Med Commun; J Nucl Med	2019; 2011	PET/CT	98 cases; 41 cases	Predicting outcome	Visual analysis; SUV_max_; SUV_max_/SUV_liver_	-	High SUV_max_ was more prevalent among patients with less favorable clinical and pathological features including unfavorable primary site, alveolar pathology, presence of regional or distant metastasis, and high-risk group. Higher SUV_max_ was significantly related to the presence of regional or distant metastasis with worse prognosis.

*-, not provided*.

*SUV_max_, maximum standardized uptake; RMS, rhabdomyosarcoma; ARMS, alveolar rhabdomyosarcoma; CIMs, conventional imaging modalities; OS, overall survival; PFS, progression-free survival; LC, local control; LRFS, local relapse-free survival*.

With regard to the staging and restaging, [^18^F] FDG PET/CT was consistently somewhat better than conventional imaging at identifying unknown primary sites, nodal involvement, and distant metastases, especially bone metastases in RMS (8–12). The primary sites of the RMS are distributed in the whole body, from head to limbs, with relatively large size at diagnosis and poor borders with surrounding tissue, which is not amenable to up-front complete surgical resection of the primary tumor and results in gross residual disease at the initiation of chemotherapy. In our study, the primary lesions only in three patients were limited to the primary sites, and other patients suffered from lymph nodes and/or distant metastases. Lymph node metastases were seen in eight patients in our study, mainly distributing to the neck, mediastinum, hilum, abdomen, and pelvis. In the previous study, approximately 15% of patients with RMS have distant metastases at diagnosis (13), and the molecular hallmark of which is paired box 3 (PAX3) or PAX7 gene fusion with forkhead box protein O1 (FOXO1) (4). According to our study, bone was the most common involved sites in metastatic RMS, and the lung ranked second. One patient (case 1) showed liver metastases in our study, and breast (14, 15) and small bowel metastasis (16) were seen in previous studies.

Complete response at the end of therapy means favorable outcome for malignant tumors. By contrast, patients with non-complete response at the end of therapy predicts poor outcome. Evaluation of therapy response in terms of FDG activity is superior to that based on the radiological performance, which may ignore the activity of tumor. Residual masses at the end of therapy may be reactive scar tissue, mature RMS that occurs as a result of differentiation of RMS cells during therapy, or residual viable tumor (6). Either scar tissue or mature RMS is non-malignant without and malignant behavior, which would not need additional therapy immediately. The data referring to the role of [^18^F] FDG PET/CT evaluation of therapy response in RMS are limited (17, 18); worse, the roles of volume-based and intensity-based PET parameters including MTV and TLG were not available for the evaluation of therapy response.

In our study, we observed an inspiring phenomenon that some patients even diagnosed as RMS with multiple lymph nodes and distant metastases would experience a complete response by the end of therapy. By analyses, the clinical characteristics including age, gender, TNM stage, therapy approaches, and SUV_mean_ level were not associated with the therapy response at the end of therapy. SUV_max_, SUV_peak_, MTV, and TLG on baseline PET were significant predictors for differentiating CMR from non-CMR. Lower SUV_max_, SUV_peak_, MTV, and TLG derived from the baseline PET were more likely to receive CMR after therapy (Table 3). SUV_max_ of primary sites of RMS was quite different in previous studies, in which it ranged from 0 to 32 (9, 11, 17–22, 24). High SUV_max_ was more prevalent among patients with less favorable clinical and pathological features including unfavorable primary site, alveolar pathology, presence of regional or distant metastasis, and high-risk group, relating to worse prognosis (23). However, there exists criticism in using SUV to evaluate therapy response, since it is calculated only from one voxel of the ROI. SUV_max_ only represents the highest metabolic activity within tumor lesions and easily affected by noise. SUV_mean_ is calculated from the average SUV value of the entire tumor, and SUV_peak_ is the mean SUV of a 1-cm^3^ three-dimensional ROI showing the highest value in the representative lesion. These factors hardly reflect the internal metabolic characteristics of tumor. MTV refers to the volume of tumor tissue, and TLG represents the metabolic activity and metabolic volume of the tumor tissue. Thus, compared to SUV, MTV and TLG have advantages in reflecting the tumor metabolic burden, which could provide a more accurate evaluation of treatment response.

Metabolic parameters derived from baseline PET, such as SUV, MTV, and TLG, are known to be applicable for evaluating therapy response in many malignant tumors. In advanced non-small-cell lung carcinoma, patients with higher values of MTV and TLG had higher probability of disease progression compared to those patients presenting with lower values, while SUV_max_ did not show a correlation with progressive disease (PD) status. MTV also resulted in being significantly different among partial response (PR), stable disease (SD), and PD groups, while SUV_max_ was confirmed to not be associated with response to therapy (25). Besides, some papers have correlated metabolic parameters derived from the baseline PET to metabolic therapy response. In locally advanced cervical cancer, patients with higher values of MTV and TLG had a higher probability of non-CMR compared to those patients presenting with lower values, while SUV_max_ was confirmed to not be associated with response to therapy (26). Similar findings were available in the mantle cell lymphoma (27). However, Voglimacci et al. (28) found that higher cervical SUV_max_ and TLG were significantly associated with poor response to chemoradiotherapy. Nakajo et al. (29) reported that the positive and negative predictive values for non-responders in esophageal cancer treated by chemoradiotherapy were 77 and 69% in MTV and 76 and 100% in TLG, respectively. On balance, SUV and other PET/CT metabolic parameters require further prospective investigation to help tailoring of treatment.

Our study has some limitations: first, the major limitation of our study is the small sample size, including only 13 patients, which limits the statistical power of our data for a definite conclusion and hardly provides cutoff value of differentiating CMR from non-CMR. Therefore, in the future, if enough cases of RMS are available, it would be given. Second, patients in our study did not receive the same treatment approaches, which would influence the final findings of this study. Third, the present study would not be able to provide the relationship of initial FDG uptake and survival because the follow-up periods were too short to reliably assess survival.

In summary, [^18^F] FDG PET/CT seems to be useful in staging RMS patients, and metabolic parameters extracted from baseline PET/CT have potential value in distinguishing CMR from non-CMR, which is worth to select therapy-sensitive patients. Additional prospective validation studies in a multicenter cohort are required for further understanding of the role of metabolic parameters extracted from baseline PET/CT in RMS.

## Data Availability Statement

All datasets generated for this study are included in the article/supplementary material.

## Ethics Statement

The studies involving human participants were reviewed and approved by Ethics Committee of The First Hospital of Jilin University. Written informed consent to participate in this study was provided by the participants' legal guardian/next of kin. Written informed consent was obtained from the individual(s), and minor(s)' legal guardian/next of kin, for the publication of any potentially identifiable images or data included in this article.

## Author Contributions

RS and FG designed the study. RS, DL, and SH participated in patient data acquisition. RS, HZ, and QL analyzed scans and data. RS and FG wrote and revised the draft. All authors read and approved the final manuscript.

## Conflict of Interest

The authors declare that the research was conducted in the absence of any commercial or financial relationships that could be construed as a potential conflict of interest.
